# Urban-Scale Chikungunya Risk Mapping in the Western Guangdong-Hong Kong-Macao Greater Bay Area Using Remote Sensing

**DOI:** 10.3390/ijerph23060730

**Published:** 2026-05-30

**Authors:** Yufeng Liu, Suhong Liu

**Affiliations:** 1 Faculty of Geographical Science, Beijing Normal University, Beijing 100875, China; 202311998046@mail.bnu.edu.cn; 2 Department of Geography, Faculty of Arts and Sciences, Beijing Normal University, Zhuhai 519087, China

**Keywords:** Chikungunya fever, Guangdong-Hong Kong-Macao Greater Bay Area, remote sensing, Mosquito Habitat Suitability Index (MHSI), spatial heterogeneity

## Abstract

**Highlights:**

**Public health relevance—How does this work relate to a public health issue?**

- This study addresses the significant public health threat posed by Chikungunya fever, specifically focusing on the large-scale local outbreak that occurred in 2025 within the densely populated western Guangdong-Hong Kong-Macao Greater Bay Area.
- It tackles the challenge of identifying mosquito-borne disease risks in complex urban environments, where rapid urbanization creates microhabitats that are difficult to monitor using traditional entomological methods.

**Public health significance—Why is this work of significance to public health?**

- The research establishes a reproducible, high-resolution (10 m) Mosquito Habitat Suitability Index (MHSI) framework that links Earth observation variables with sub-city outbreak records to characterize urban-scale environmental suitability.
- It further shows that environmental suitability alone cannot fully account for observed spatial mismatch and that transportation, activity spaces, and healthcare-related human processes are important complementary factors.

**Public health implications—What are the key implications or messages for practitioners, policymakers and/or researchers in public health?**

- Provides public health authorities and policymakers with high-resolution spatial evidence that may help identify areas warranting closer surveillance and more targeted vector-control attention.
- Advises researchers that future infectious disease risk models should move beyond purely environmental-climatic metrics by more explicitly integrating human mobility, urban spatial organization, and healthcare accessibility.

**Abstract:**

This study presents a reproducible high-resolution framework for assessing urban chikungunya environmental suitability and outbreak-related spatial heterogeneity during the 2025 outbreak in the western Guangdong–Hong Kong–Macao Greater Bay Area. Using Sentinel-2–derived environmental indicators together with a random forest–based residual correction of Landsat surface temperature, we developed a 10 m weighted additive Mosquito Habitat Suitability Index (MHSI). Index weights were empirically derived by comparing reported case locations at the street and town level with randomly sampled background points. The optimized weighting scheme indicated that humidity- and water-related conditions contributed more strongly to habitat suitability than vegetation and temperature. Reported case locations generally corresponded to higher MHSI values than background locations, suggesting that the index captures broad spatial patterns of environmental suitability. Comparison with a coarser, model-derived global chikungunya risk map was used as an external comparative consistency assessment rather than predictive validation, showing moderate agreement at the macro-spatial scale (Pearson *r* = 0.3421) after correction for spatial autocorrelation. Residual-difference analysis, combined with multiple points-of-interest (POI) categories, ordinary least squares (OLS), and geographically weighted regression (GWR), further suggested that human activity, transport connectivity, and healthcare accessibility may account for part of the remaining spatial mismatch not explained by environmental suitability alone. Sensitivity analyses indicated that the broad LST downscaling pattern and the exploratory GWR interpretation were reasonably stable under alternative sampling, smoothing, grid-size, and bandwidth settings. Taken together, this framework provides preliminary spatial evidence for high-resolution environmental suitability assessment and exploratory interpretation of outbreak-related spatial heterogeneity, while underscoring the need for finer-scale epidemiological data and more explicit representation of human-driven processes.

## 1. Introduction

Chikungunya fever is an acute febrile illness caused by chikungunya virus (CHIKV) and transmitted by Aedes mosquitoes. Clinically, it is characterized by abrupt onset of high fever, rash, and severe, often persistent polyarthralgia [1]. Historically, the disease was largely restricted to sylvatic transmission cycles in Africa and Asia [2], but since 2004 it has undergone a marked global resurgence and now poses a substantial public health burden [3]. Microevolutionary changes in the viral genome, including the A226V point mutation, have significantly increased viral fitness in Aedes albopictus by enhancing infection, replication, and transmission within the mosquito midgut [4]. This adaptive advantage has allowed CHIKV to reduce its reliance on Aedes aegypti and establish local transmission cycles in temperate and subtropical regions where Aedes aegypti is absent but Aedes albopictus populations are abundant [5].

Globally, Aedes albopictus and Aedes aegypti are recognized as the principal vectors of CHIKV [6]. Aedes albopictus is especially notable for its broad ecological tolerance and strong association with human-modified environments, traits that have contributed to its remarkable invasive success worldwide [7]. In transitional climatic zones between the temperate and subtropical regions, including southern China, it has increasingly displaced Aedes aegypti as the dominant vector species [8]. Chikungunya also has a high attack rate, often affecting 30–50% of the population in immunologically naïve settings [9]. Although case fatality is generally low, the persistent joint pain associated with infection can substantially impair work capacity and quality of life, and the resulting long-term economic burden is often underestimated [10].

Mosquito population dynamics are strongly shaped by habitat conditions. During rapid urbanization, the expansion of impervious surfaces and intensification of the urban heat island effect create abundant breeding opportunities for Aedes albopictus [11]. Discarded artificial containers, vacant land, and fragmented vegetated shade together form microhabitats that favor both larval development and adult survival [12]. At the climatic scale, temperature directly affects transmission efficiency by altering the extrinsic incubation period, biting frequency, and adult survival of mosquitoes [13]. The nonlinear relationship between temperature and vector-borne transmission further suggests that the thermal optimum for viral spread often lies below the upper thermal threshold for mosquito survival [14]. At the same time, shifts in precipitation patterns influence both the abundance and spatial distribution of potential breeding sites [15].

Traditional entomological surveillance indices provide direct measures of vector density [16], but they are costly and difficult to sustain continuously at large spatial scales, often leaving gaps in early warning. As a result, current research has increasingly shifted toward risk-mapping frameworks based on Maximum Entropy (MaxEnt) and other ecological niche models (ENMs) [17]. These approaches estimate potential habitat suitability by integrating meteorological, environmental, and socioeconomic variables [18]. In doing so, they have helped clarify how human mobility and environmental conditions interact to shape transmission risk and have been used to project the poleward expansion of mosquito vectors under climate change [19]. More recently, some studies have incorporated machine-learning methods to analyze high-dimensional remote sensing data, further improving the accuracy of risk prediction [20,21].

Remote sensing (RS), with its synoptic coverage and multi-temporal observational capacity, has become an indispensable tool in vector biology [22], providing continuous environmental data across spatial scales through the retrieval of surface parameters [23]. Early applications relied largely on coarse-resolution imagery to derive vegetation and temperature indices and to examine the constraints imposed by broad climatic zones [24]. Among specific metrics, multispectral RS indices have proved particularly useful for characterizing habitat suitability [25]. For example, vegetation structural heterogeneity has been positively associated with Aedes mosquito density [26]; water indices such as Normalized Difference Water Index (NDWI) are widely used to delineate wet environments closely linked to oviposition and larval development [27]; and land surface temperature (LST) retrieval is effective for capturing the nonlinear influence of urban heat islands on mosquito life cycles, showing that urban cores often support longer periods of vector activity [22,28,29].

The 2025 chikungunya outbreak in Guangdong did not emerge simultaneously across the province, but instead followed a clear spatiotemporal sequence, beginning with intense transmission in Foshan, then spreading to neighboring cities, and eventually expanding across the western Greater Bay Area. According to China Centers for Disease Control and Prevention (CDC) Weekly, Foshan reported a clustered outbreak on 9 July 2025, with the earliest confirmed symptom onset traced to 16 June. By 26 July, Guangdong had recorded 4824 confirmed cases, of which 4754 (98.5%) were reported in Foshan, including 4208 in Shunde District alone. Information subsequently cited by the Hong Kong SAR Government from the Foshan Municipal Health Commission further indicated that, as of 20 July, most cases in Foshan were concentrated in a small number of highly connected towns in Shunde, particularly Lecong, Beijiao, and Chencun, whereas Chancheng and Nanhai reported substantially fewer cases. This pattern suggests that the epidemic did not initially diffuse evenly across urban space, but instead began as intense local clustering within a limited number of connected township- and street-level built-up areas. China CDC also characterized the event as a locally transmitted outbreak initiated by an imported infection, and the first imported case reported in Macao on 18 July had visited Shunde during the incubation period, lending further contextual support to a process of importation, local amplification, and subsequent spillover. The epidemic then began to extend to surrounding cities. Provincial notifications covering 27 July to 2 August reported 2892 new local cases, including 2770 in Foshan, 11 in Zhongshan, 6 in Jiangmen, and 4 in Zhuhai, indicating that although Foshan remained the dominant epidemic center, signs of local transmission had already emerged elsewhere in the western Greater Bay Area. At a later stage, intercity differences became even more pronounced: Jiangmen reported its first case on 16 July and had accumulated 1714 cases by 19 September, while World Health Organization (WHO) summaries indicated that by 27 September Guangdong had recorded 16,452 cumulative locally confirmed cases, including 10,032 in Foshan, 5209 in Jiangmen, 590 in Guangzhou, 128 in Shenzhen, 60 in Zhuhai, and 54 in Zhongshan. Taken together, these observations indicate that the 2025 outbreak in Guangdong was spatially concentrated primarily in Foshan and Jiangmen, and temporally characterized by early escalation in Foshan, subsequent growth in Jiangmen, and lower-level spillover to cities such as Zhuhai and Zhongshan. This spatiotemporal progression suggests that risk variation across the study area was shaped not only by underlying environmental suitability, but also by imported-case initiation, the continuity of township-scale built-up space, intercity commuting linkages, and variation in case detection and reporting capacity.

Against this outbreak backdrop, three closely related research gaps remain. First, although global and regional chikungunya risk models are valuable for identifying broad gradients of suitability, their spatial resolution is generally too coarse to capture neighborhood-scale heterogeneity within densely connected urban agglomerations. Second, even within urban remote-sensing studies, few frameworks integrate 10 m habitat-related indicators with empirically derived, case-informed weighting during a specific local outbreak. Third, environmental suitability alone cannot fully account for urban transmission dynamics, yet residual spatial mismatches are rarely interpreted in conjunction with human-process proxies such as transport connectivity, residential activity spaces, outdoor exposure environments, and healthcare accessibility. To address these gaps, this study develops a reproducible 10 m MHSI workflow, derives index weights from observed 2025 sub-city case locations relative to environmental background samples, and uses residual mismatch against an external comparative surface to explore where human-process proxies may help explain spatial variation beyond environmental suitability alone.

## 2. Materials and Methods

### 2.1. Research Area

**Figure 1:**
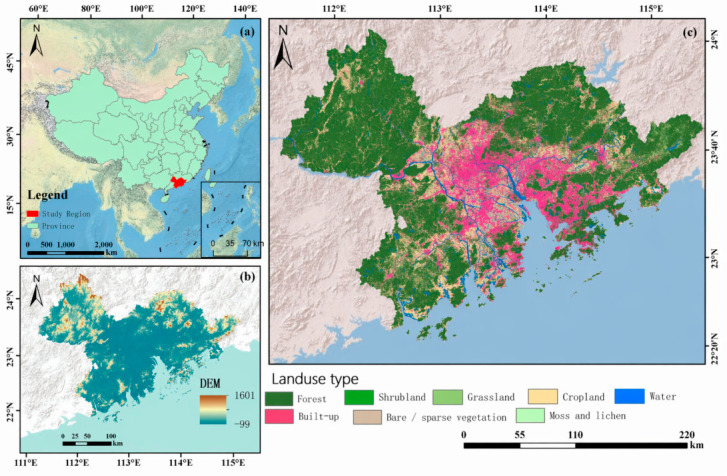
Map of study area of the western Guangdong–Hong Kong–Macao Greater Bay Area: (**a**) national location; (**b**) regional location within the Greater Bay Area; and (**c**) administrative divisions and major cities. (Vector data source: https://cloudcenter.tianditu.gov.cn/).

The western Greater Bay Area (21–23° N, 112–114° E) spans approximately 15,000 km^2^ with a population exceeding 20 million (Figure 1). Foshan and Zhongshan serve as industrial hubs featuring mixed urban-rural landscapes, while Jiangmen and Zhuhai possess coastal wetlands and mangrove forests. The subtropical climate (annual average temperature 22–25 °C, rainfall 1500–2000 mm) favors Aedes mosquito breeding. Urban expansion has increased stagnant water and vegetation fragments, amplifying vector risks.

### 2.2. Data Source

#### 2.2.1. Remote Sensing Data and Processing

High-resolution environmental variables for the western Guangdong–Hong Kong–Macao Greater Bay Area were generated on the Google Earth Engine (GEE) platform for the exposure window from 1 March to 31 May 2025 [30]. Surface characteristics, including vegetation, water bodies, and built-up areas, were derived from the Sentinel-2 Level-2A surface reflectance product (COPERNICUS/S2_SR_HARMONIZED; European Space Agency, Paris, France), accessed and processed on the Google Earth Engine platform (Google LLC, Mountain View, CA, USA) [31]. Images were first filtered by study area and acquisition date within the exposure window. Cloud and cirrus contamination were removed using the QA60 bitmask (bits 10 and 11), and reflectance values were rescaled to a uniform 0–1 range. Four spectral indices—NDVI, NDWI, NDBI, and EVI—were then calculated for each scene. To generate a representative environmental surface for the study period, these index layers were composited using the median and clipped to the study area.

**Figure 2:**
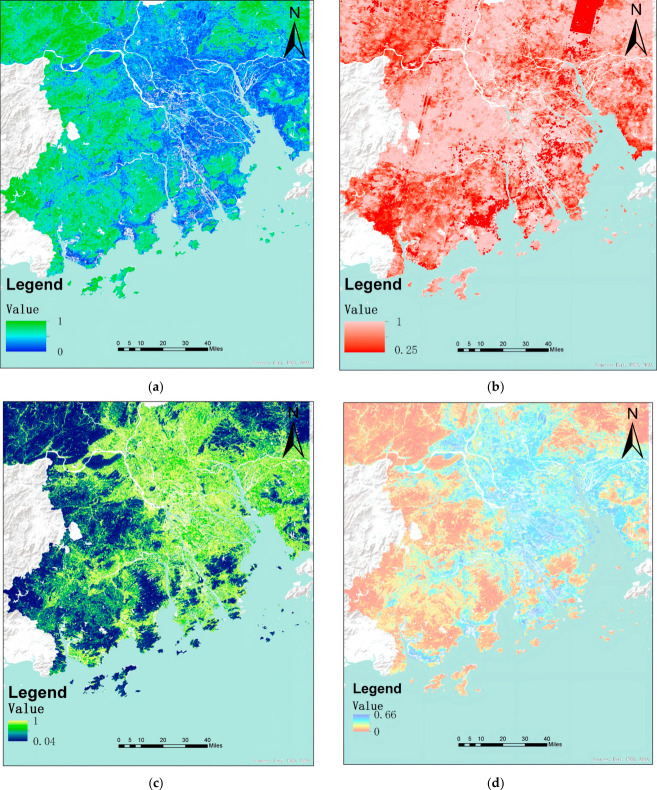
Spatial distribution of environmental factors: (**a**) Humidity, (**b**) Temperature, (**c**) Vegetation, and (**d**) Water bodies.

The environmental exposure window was defined as 1 March to 31 May 2025 to capture antecedent conditions preceding the main phase of local transmission. This lagged period was chosen to reflect the time required for mosquito development, viral extrinsic incubation, human incubation, and subsequent case detection and reporting. In the revised framework, empirical positive samples were derived from publicly reported sub-city case locations released during the 2025 outbreak, rather than from weekly case counts aggregated at the city level. The March–May environmental composites were therefore used to represent the background habitat conditions likely to shape early outbreak suitability, while the case-location dataset reflected the broader period of public reporting during the 2025 epidemic. This temporal alignment should be interpreted as an epidemiologically informed approximation rather than a strict reconstruction of event-specific exposure. Because the environmental composites were constructed for March–May 2025, their relevance may be stronger for the early phase of the outbreak than for cases reported later in September–November. Seasonal changes during the monsoon period, including variation in surface moisture, NDWI, and land surface temperature, may have altered local habitat suitability and weakened the correspondence between the static MHSI surface and later-phase case locations (Figure 2).

To characterize intra-urban thermal heterogeneity while preserving spatial consistency with the 10 m Sentinel-2 environmental indicators, we adopted a statistical downscaling approach based on random forest regression with residual correction. The aim was not to generate a physically independent 10 m thermal infrared observation, but rather to derive a spatially refined temperature surface more compatible with the scale of the environmental predictors used in subsequent MHSI construction.

Landsat Collection 2 Level-2 surface temperature products from Landsat 8 [32] and Landsat 9 [33] were used as the thermal reference for the exposure window from 1 March to 31 May 2025. Clouds, cloud shadows, and snow were masked using the QA_PIXEL band, which provides pixel-level quality flags for contaminated observations, and the ST_B10 band, which contains the scaled land surface temperature retrieval, was converted to degrees Celsius. A median composite was then generated to produce a representative 30 m land surface temperature for the study period.

Random forest regression was selected because the relationship between land surface temperature and land-cover characteristics in urban environments is often nonlinear and shaped by interactions among vegetation, water-related surface conditions, and built-up intensity. Compared with simple linear resampling or parametric regression, random forest can capture these empirical relationships flexibly without requiring a predefined functional form, making it well suited to statistical downscaling in heterogeneous urban landscapes.

Three Sentinel-2–derived indices—NDVI, NDWI, and NDBI—were used as predictors. These variables were chosen because they represent three key surface dimensions known to influence urban thermal conditions: vegetation cover, moisture or water-related surface characteristics, and built-up intensity. To ensure scale consistency during model training, the Sentinel-2 indices were first resampled and reprojected to the 30 m Landsat grid. We then generated 3000 random points across the study area and extracted the corresponding LST and predictor values to construct the training dataset. The random forest model was trained using 200 trees, a minimum leaf population of 2, a bag fraction of 0.7, and a fixed random seed of 42.

After model fitting, a 30 m predicted temperature surface ($\hat{LST}$_30_) was generated, and the residual field was calculated as R_30_ = LST_30_ − $\hat{LST}$_30_. Residual correction was introduced because the regression model may not fully preserve the broader thermal background inherited from the Landsat observations. Rather than assuming that the residuals were spatially independent, we treated the residual field as containing both local noise and spatially structured bias. A Gaussian kernel smoothing procedure (radius = 1.5 pixels) was therefore applied to the 30 m residual field to extract its low-frequency, spatially coherent component while reducing pixel-level noise. The smoothed residual surface was then resampled to 10 m.

In parallel, the trained random forest model was applied to the original Sentinel-2 predictor layers at 10 m to generate a fine-scale predicted temperature field ($\hat{LST}$_10_). The final downscaled temperature surface was obtained through residual backfilling: LST_10_ = $\hat{LST}$_10_ + R_10_. This strategy allows the 10 m prediction to reflect local land-cover heterogeneity while preserving the broader thermal baseline represented by the Landsat observations. In other words, the random forest component contributes spatial detail, whereas the smoothed residual field helps retain consistency with the original thermal reference.

Model performance was evaluated using a 70/30 hold-out validation strategy. Agreement between observed and predicted 30 m LST in the test set was assessed using the coefficient of determination (R^2^), mean absolute error (MAE), root mean square error (RMSE), and mean bias error (MBE). The hold-out validation yielded R^2^ = 0.72, MAE = 0.92 °C, RMSE = 1.79 °C, and MBE = 0.37 °C, indicating that the model reproduced the main spatial variation in the Landsat-based thermal field with a small positive bias. To further evaluate spatial dependence in the residual field used for residual correction, a sample-based global Moran’s I statistic with a fixed distance-band binary weighting scheme was calculated (sample size = 800; distance threshold = 1000 m). The raw 30 m residual field showed positive spatial autocorrelation (I = 0.114), and the smoothed residual field also retained positive spatial structure (I = 0.082), with both values exceeding the expected value under spatial randomness (E[I] = −0.0013).

The downscaled LST product should therefore be interpreted as a statistically refined temperature field rather than as a physical enhancement of native thermal infrared resolution. This approach assumes that the empirical relationship between LST and the selected surface-cover predictors is sufficiently stable within the study area and that the interpretable component of the residual field is mainly expressed as low-frequency spatial bias. The Moran’s I results indicate that residual errors were spatially structured rather than purely random, supporting the use of a smoothed residual correction to retain low-frequency spatial bias, but not implying that the residual field is fully characterized by the present smoothing procedure. To examine the stability of key implementation choices, we further varied the number of random training points (1000, 3000, 5000, and 10,000) and the Gaussian smoothing radius (0.5, 1.5, and 3.0 pixels). Across the 12 sensitivity settings, hold-out R^2^ ranged from 0.6307 to 0.6899, MAE from 0.2845 °C to 0.3046 °C, RMSE from 0.3783 °C to 0.3939 °C, and MBE from −0.2202 °C to 0.2457 °C. The alternative 10 m LST surfaces were also highly correlated with the baseline surface (sample-based spatial correlation = 0.9985–0.9997), indicating that the broad thermal pattern was not strongly dependent on the specific choice of 3000 training points or the 1.5-pixel Gaussian smoothing radius. Accordingly, the downscaled 10 m LST layer is best regarded as an operational statistically refined environmental proxy for subsequent suitability mapping rather than as an independent observed temperature product, and its uncertainty should be carried conceptually into the interpretation of the downstream MHSI results.

#### 2.2.2. Clinical Data

Clinical data were compiled from a manually curated public dataset of chikungunya-related records for the Guangdong–Hong Kong–Macao Greater Bay Area in 2025. The full database comprised 218 geocoded sub-city records, including entries on case distribution, risk areas, and mosquito density. Primary sources included official government and public health releases from the Macao Health Bureau, Hong Kong Government News, municipal government pages in Jiangmen, and CDC releases or government webpages from Dongguan, Zhuhai, and Shenzhen. These were supplemented by government-affiliated local media and major news platforms that explicitly reported or relayed official outbreak information, including Guanhai Media, Southern Plus, Dongguan Time, Shenzhen News, and Xinhua. For empirical estimation of MHSI weights and internal consistency assessment, we used the 96 records classified as case distribution as positive samples. These case-location records spanned event dates from 6 June to 13 November 2025 and public release dates from 11 June to 3 December 2025. Records were included only if they referred to chikungunya-related events in 2025 within the Greater Bay Area and could be geolocated to at least the level of a street or town, community, or named site; province-level or city-level summaries without usable sub-city locational information were excluded. Geocoding was conducted on a best-effort basis, using street- or town-level administrative center points wherever possible, with district or city centers used only when finer spatial detail was unavailable. During data compilation, exact duplicate extractions were removed, whereas repeated mentions of the same street or town across different reports or reporting dates were retained when they represented distinct public records or source chains. When multiple news pages simply reposted the same official notice without adding new locational information, they were consolidated where possible; however, when reposted pages provided the only accessible public trace for a given location or reporting context, the source chain was retained in the source_name, source_url, and note fields.

Because the dataset was derived from publicly disclosed reports rather than from individual surveillance records, it does not represent a complete census of all infections. In addition, the reported locations refer to street/town, community, or publicly named sites rather than precise household addresses. Therefore, the clinical dataset was used as a pragmatic sub-city outbreak reference for weight construction and internal evaluation rather than as a full case registry of the outbreak.

#### 2.2.3. Verified Case Data

**Figure 3:**
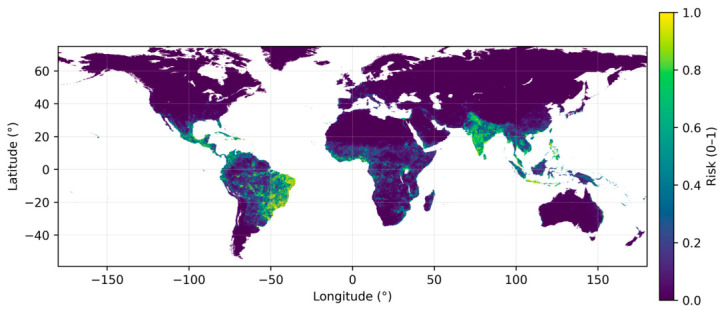
Global chikungunya risk map used as the external comparative reference surface.

As Figure 3 shows, Verified case data are sourced from published global arbovirus occurrence databases, supplemented by integrating multiple public outbreak surveillance and notification channels to complete temporal and spatial records. These include ProMED [34] (primarily filling gaps for chikungunya and Zika from 2015 to 2022), European Centre for Disease Prevention and Control (ECDC) notifications [35], World Health Organization (WHO) [36] regional outbreak updates, and HealthMap [37] outbreak capture data. To mitigate biases from varying reporting frequencies and duplicate submissions across regions, raw records were maintained in both point and administrative polygon spatial formats. Duplicate entries for the same location (or administrative unit) underwent spatial thinning. Newly added HealthMap information underwent cross-verification against peer-reviewed literature and official reports to enhance the reliability and consistency of case occurrence data.

#### 2.2.4. Sources of Cultural POI Data

Points-of-interest (POI) data representing human activity environments were obtained via the “Polygon Range Query” interface (/v3/place/polygon) from the AutoNavi Map Open Platform (AMap; AutoNavi Software Co., Ltd., Beijing, China) [38]. POI themes were classified and retrieved using keyword groups, including healthcare services (hospitals, community health centers, clinics, and pharmacies), population gathering and residential activities (residential areas, schools, shopping malls, markets, restaurants, industrial parks, etc.), transportation flow (stations, subways, airports, ports, hotels/lodging), and outdoor exposure sites (parks, wetlands, water bodies, etc.). Search results extracted the POI unique ID, name, category code, address, and latitude/longitude. After deduplication by POI ID, point feature data (EPSG:4326) were generated and exported as separate CSV files for subsequent spatial analysis.

### 2.3. Research Methods

#### 2.3.1. MHSI

The study used an optimized multi-factor composite weighting model to calculate the Mosquito Habitat Suitability Index (MHSI) [39], with the aim of refining the assessment of environmental support for mosquito breeding across the region. The underlying premise is that mosquito survival and reproduction are shaped not by any single environmental variable, but by the combined influence of multiple dimensions, including water availability, vegetation conditions, thermal background, and humid or shaded microhabitats. We adopted an additive formulation because the purpose of the MHSI was to characterize overall environmental suitability for mosquito habitat at the urban scale, rather than to represent a strictly mechanistic model of transmission. In heterogeneous urban settings, different environmental dimensions may contribute jointly to habitat suitability, and the influence of one component does not necessarily vanish when another is relatively weak. An additive composite structure therefore offers an interpretable and reproducible means of integrating multiple environmental proxies while allowing partial compensation among factors across complex urban landscapes.

Regarding environmental factor selection and index construction, MHSI is designed as a combination of four key ecological drivers: Water Proximity (W), Vegetation Suitability (V), Temperature Suitability (T), and Humidity/Canopy Cover Suitability (H). Specifically, NDWI serves as an indicator of water proximity, NDVI characterizes general vegetation cover and vegetation-associated resting conditions, LST represents the thermal background for mosquito vectors and viral growth, and EVI is used as a proxy for moist vegetation, canopy closure, and shaded microhabitats rather than as a direct measure of humidity. These variables are not assumed to be strictly independent; instead, they were chosen to capture different but partially overlapping dimensions of environmental support for mosquito breeding and survival. To mitigate the impact of extreme values and unify the dimensions of different indices, the spectral indices and temperature were linearly normalized within the 1–99 percentile range, compressing them into the [0, 1] interval.

The multi-factor habitat suitability index for each grid cell is defined as Equation (1).

(1) MHSI=W⋅a+V⋅b+T⋅c+H⋅d,

In Equation (1), a, b, c, and d represent the weight parameters to be estimated, reflecting the relative contributions of different environmental factors to overall habitat suitability. The constraint a + b + c + d = 1 is applied to ensure that the weights have clear proportional interpretation.

#### 2.3.2. Empirical Weight Estimation Using Observed Case Locations

The additive MHSI was defined as a weighted combination of the four environmental components. To estimate these weights empirically, reported chikungunya case locations were treated as positive samples, and randomly generated background locations within the study area were used as environmental reference samples. The background points were not interpreted as confirmed non-case locations; rather, they represented the environmental background available across the study area.

For each optimization run, 96 background points were randomly sampled from valid land pixels within the study-area mask, matching the number of reported case-location records and producing a balanced 1:1 case-background comparison. Valid pixels were required to have finite values for all four MHSI components and were screened using the same mask applied during MHSI construction, which excluded pixels with NDWI > 0, NDVI < 0, or population = 0. Background points were used to represent available environmental conditions and were not interpreted as confirmed absence locations. The sampling and weight optimization procedure was repeated 100 times using a fixed random seed of 42. For each candidate weight combination satisfying the above constraints, MHSI values were extracted at case and background locations, and discriminatory ability was quantified using the area under the receiver operating characteristic curve (AUC). The final weights were summarized using the median values across the 100 repeated background resamplings.

Because the reported locations are still derived from public disclosures rather than a complete surveillance registry, this step was treated as an empirical tuning procedure for the composite index rather than as a definitive ecological parameter estimation. After the weights had been determined, the final MHSI surface for the entire study area was reconstructed using the optimized additive combination of the four environmental components. This surface was then used for subsequent internal case-based consistency assessment, residual spatial diagnosis, and comparative analysis with the external reference risk map. Importantly, because the same public case-location dataset contributed to weight estimation, the internal case-based assessment reported later should be read as a sanity check on spatial consistency rather than as an independent test of predictive performance.

#### 2.3.3. External Comparative Reference and Risk Map–MHSI Difference Definition

The external chikungunya risk map was used as a model-derived comparative reference surface rather than as an observed ground truth. In this study, the residual-difference field was defined as the pixel-level difference between the external risk map and the locally derived MHSI surface (risk map−MHSI), not as a residual between observed cases and model predictions. Positive values therefore indicate locations where the external comparative surface assigns higher relative risk than the local environmental suitability index, whereas negative values indicate locations where the local MHSI is higher than the external reference. This field was interpreted as a diagnostic of representational mismatch between two risk-related surfaces. Moran’s I was then used to examine whether this mismatch retained spatial structure, thereby motivating exploratory analysis of additional human-process proxies.

Global Moran’s I was calculated as:

(2) $$I=\frac{n}{S_{0}}\cdot \frac{\sum_{i=1}^{n}\sum_{j=1}^{n}w_{ij}(x_{i}-\overset{-}{x})(x_{j}-\overset{-}{x})}{\sum_{i=1}^{n}(x_{i}-\overset{-}{x}{)}^{2}}$$

where $x_{i}$ is the residual value in spatial unit *i*, $\overset{-}{x}$ is the mean residual, $w_{ij}$ is the spatial weight between units i and j, and $S_{0}$. The expected value under spatial randomness is:

(3) $$E(I)=-\frac{1}{n-1}$$

In this study, Moran’s I was computed for both the raw and smoothed residual fields using a sample-based implementation with a fixed distance-band weighting scheme. This diagnostic was used to determine whether residual variation remained spatially structured after construction of the suitability surface and residual smoothing.

#### 2.3.4. Geographically Weighted Regression of Residuals on Major POI Groups

To move beyond descriptive POI overlay, this study further used a global ordinary least squares (OLS) model and a geographically weighted regression (GWR) model to quantify the relationship between residual intensity and major POI groups. Here, residual intensity refers to the grid-level value of the residual-difference field defined above (risk map−MHSI). The rationale was that, after comparing the local MHSI surface with the external comparative reference, the remaining spatial mismatch may partly reflect human-process factors not captured by environmental suitability alone. Four major POI groups were considered: Medical, Crowd Aggregation, Mobility and Importation, and Exposure scenarios.

The study area was divided into regular 3 km × 3 km grid cells in UTM Zone 49N (EPSG:32649). For each grid cell, the mean residual value and the counts of the four POI groups were calculated after aggregating residual sample points and POIs to the same grid. Before model fitting, predictor counts were transformed using log(1 + x) to reduce skewness, and cells with fewer than two total POIs across the four major groups were excluded to avoid extremely sparse units. A global OLS model was first fitted as:

(4) $$y_{i}=\beta_{0}+\sum_{k=1}^{p}\beta_{k}x_{ik}+\varepsilon_{i}$$

In Equation (4), yi is the mean residual-difference value in grid cell i, xik is the transformed count of POI group k, βk is the corresponding global coefficient, and εi is the error term. During preprocessing, zero-variance predictors were removed, and pairwise collinearity was screened using a |r| > 0.90 threshold before the GWR analysis was fitted.

Because the effects of human activity spaces are unlikely to be spatially stationary in a heterogeneous urban agglomeration, a geographically weighted regression was then applied as:

(5) $$y_{i}=\beta_{0}(u_{i},v_{i})+\sum_{k=1}^{p}\beta_{k}(u_{i},v_{i})x_{ik}+\varepsilon_{i}$$

In Equation (5), (ui, vi) denotes the coordinates of grid cell i, and βk(ui, vi) is the location-specific coefficient for predictor k. Local coefficients were estimated using an adaptive bi-square kernel. Bandwidth selection was performed automatically, with fallback to a larger neighbor set if the initial search failed to converge. In the final model, the automatically selected adaptive bandwidth was 122 grid cells, and the fallback procedure was not triggered. The effective number of parameters (ENP = 143.49), model degrees of freedom (1242.51), and residual degrees of freedom (1194.93) were also reported to support assessment of local model flexibility and potential overfitting. To further evaluate model stability, we repeated the GWR analysis using 2 km, 3 km, and 5 km grid cells and, for each grid size, compared the automatically selected adaptive bandwidth with bandwidths decreased and increased by 20%. For each setting, R^2^, adjusted R^2^, AICc, ENP, local R^2^, and the proportion of positive local coefficients for each POI group were recorded. In this framework, OLS was used to assess overall association, whereas GWR was used to identify spatial heterogeneity in the relationships between residual intensity and POI groups. These results were interpreted as exploratory spatial statistical support for human-related patterns in the mismatch between the MHSI and the external comparative surface rather than as direct causal proof of transmission mechanisms.

## 3. Results

The optimized weighting results reveal substantial variation in the contribution of the four MHSI components: Water Proximity (W, a) = 0.2236 (22.36%), Vegetation Suitability (V, b) = 0.1114 (11.14%), Temperature Suitability (T, c) = 0.1326 (13.26%), and Humidity/Canopy Cover Suitability (H, d) = 0.5325 (53.25%). The optimized weighting scheme achieved an AUC of 0.762 for case-background discrimination, indicating acceptable discriminatory performance under the present balanced sampling design. Overall, humidity-related and water-related components contributed more strongly to case-background discrimination than vegetation and temperature, suggesting that moisture-related environmental conditions played a comparatively larger role in shaping the composite suitability gradient under the present model settings.

**Figure 4:**
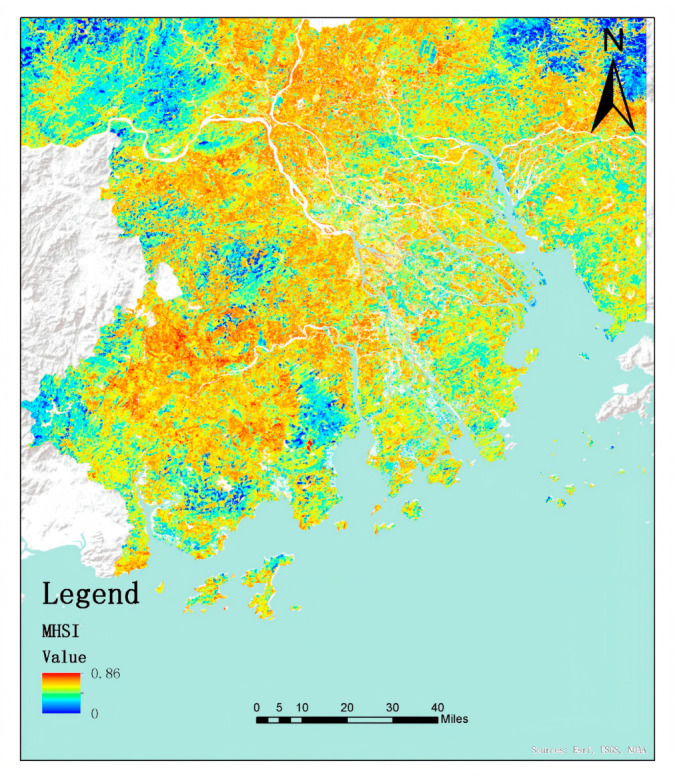
Spatial distribution of the Mosquito Habitat Suitability Index (MHSI) as an environmental suitability surface.

Figure 4 visualizes the final MHSI surface. After substituting the optimized weights into all pixels across the study area, the generated composite environmental suitability surface exhibits a distinct east-high, west-low spatial distribution pattern. High-suitability zones are primarily concentrated in the continuous built-up belt spanning the central-eastern part of the study area, as well as in the densely crisscrossed river networks of the plain convergence zone.

These areas are consistent with the stronger contribution of humidity, suitability and proximity to water bodies, forming a broad and relatively connected environmental risk base. In contrast, the MHSI in the western and peripheral areas of the study region is generally low, with high-suitability pixels mostly confined to local water systems or specific microhabitats, exhibiting a scattered and fragmented distribution pattern. Overall, this spatial mapping delineates regional heterogeneity in environmental suitability but should be interpreted as a map of environmental support for potential transmission rather than a complete representation of the full transmission process.

### 3.1. Internal Consistency Assessment Using Observed Case Locations

**Figure 5:**
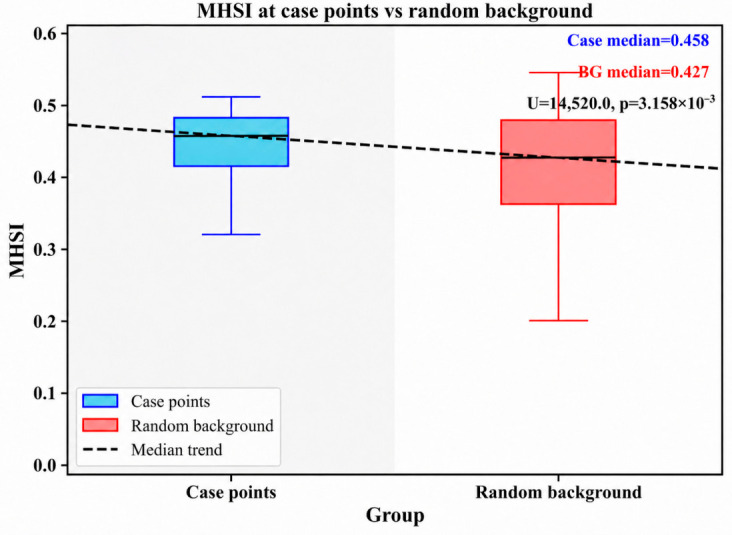
Distribution of MHSI values at actual case points and random background locations.

Figure 5 compares MHSI values extracted at observed case locations with those extracted from randomly sampled background locations within the study area. The median MHSI at case points (0.458) was higher than that at random background points (0.427), indicating that reported cases were more likely to occur in areas with elevated environmental suitability. The Mann–Whitney U test further showed that this difference was statistically significant (U = 14520.0, *p* = 3.158 × 10^−3^). These results suggest that, although MHSI is not intended to predict individual cases deterministically, the index captures the broad spatial tendency for actual chikungunya cases to be concentrated in environmentally more suitable locations. Because the same case dataset contributed to empirical weight tuning, this analysis should be interpreted as an internal consistency assessment rather than as independent external validation.

### 3.2. External Comparative Consistency Assessment

**Figure 6:**
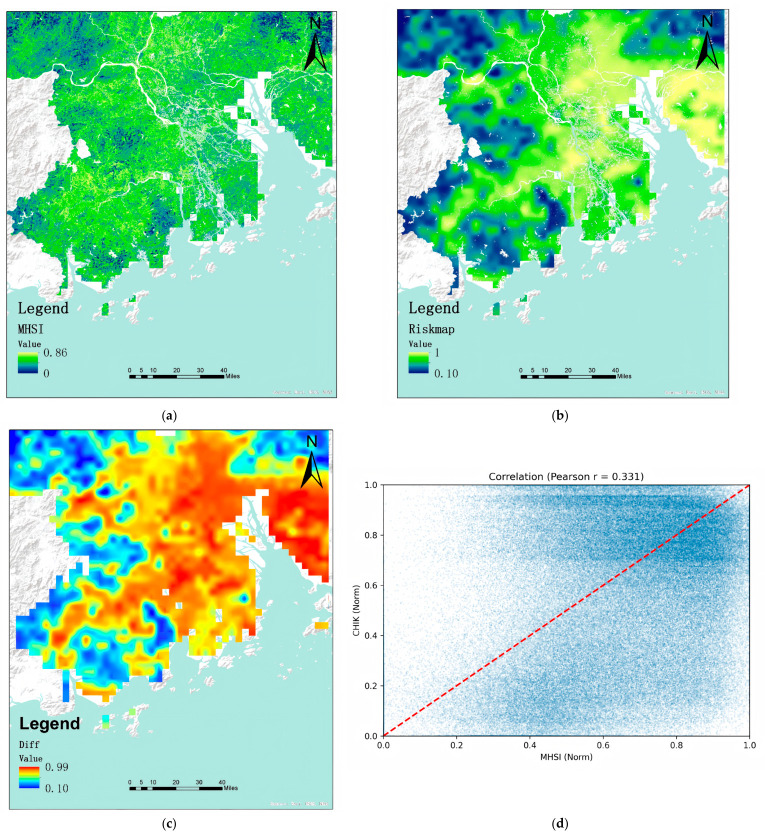
Comparison between the MHSI and the external comparative risk map: (**a**) MHSI after normalization to 0–1; (**b**) risk map after normalization to 0–1; (**c**) residual-difference map between the two surfaces; and (**d**) pixel-level Pearson correlation scatter plot.

To compare the broad spatial pattern of the MHSI developed in this study with an existing large-scale chikungunya risk product, the MHSI surface was aligned with the external comparative reference surface derived from the global chikungunya risk map, and both layers were normalized to a common 0–1 scale (Figure 6). Visual comparison, difference mapping, ordinary pixel-level correlation, and spatially corrected inference were then used to assess whether the two surfaces showed similar macro-spatial gradients. Because the reference layer is model-derived rather than observational and differs substantially in spatial resolution from the MHSI surface, this exercise should be understood as an external comparative consistency assessment, not as predictive validation or validation against ground-truth case data.

From the overall spatial distribution perspective, the MHSI surface and the comparative reference map show a broadly similar macro-scale pattern within the study area, with corresponding high-value and low-value belts across major parts of the region. This suggests that the MHSI captures part of the same broad spatial gradient represented by the global reference product. At the same time, the difference map (chikungunya risk map−MHSI) reveals systematic local departures, which are not surprising given the differences in scale, covariates, and modeling objectives between the two surfaces. These discrepancies therefore indicate differences in representation rather than direct model error against a ground-truth benchmark.

At the pixel scale, MHSI showed a moderate positive correlation with the external comparative reference values (Pearson r = 0.3421) based on 2,623,684 valid pixels, indicating that the locally derived MHSI and the external chikungunya risk map shared a consistent broad spatial gradient. To account for the fact that raster pixels are not spatially independent, we further applied three spatially corrected inference procedures. First, a modified t-test reduced the effective sample size to 11,010.13 (df = 11,008.13), yet the corrected test statistic remained highly significant (t = 36.84, *p* = 3.06 × 10^−280^). Second, a torus-shift spatial null model based on 999 two-dimensional cyclic shifts produced a null distribution centered near zero (mean = −0.0003, sd = 0.0959), whereas the observed correlation r = 0.3421 lay far in the right tail (two-sided *p* = 0.001). Third, a 999-run block permutation test using 32-pixel spatial blocks yielded a similar near-zero null distribution (mean = −0.0009, sd = 0.0136), and the observed correlation again lay far outside the null range (two-sided *p* = 0.001). Taken together, these results indicate that the MHSI–risk map agreement cannot be explained simply by shared large-scale spatial smoothness or local clustering, but remains robust after explicit correction for spatial autocorrelation. Accordingly, the reference comparison is best interpreted as evidence of externally compared macro-spatial consistency rather than as a test of predictive accuracy against independent local surveillance data.

## 4. Discussion

Although the MHSI developed in this study captures part of the observed chikungunya spatial pattern, it should be interpreted primarily as an environmental suitability surface rather than as a complete transmission-risk or case-occurrence model. The internal case-based comparison and the external risk map comparison serve different purposes: the former is an internal consistency check because the same public case-location dataset contributed to weight tuning, whereas the latter is a macro-spatial consistency assessment against a model-derived reference surface. Together, these assessments support the spatial plausibility of the framework, but they do not constitute predictive validation or causal identification.

The results also highlight the trade-offs of an outbreak-oriented framework. Compared with global or regional models, this approach provides finer urban resolution but has lower transferability. Compared with operational vector-surveillance or time-series forecasting systems, it is better suited to interpreting the spatial signature of a specific outbreak than to producing long-term predictions. The POI overlay, OLS, and GWR analyses are therefore used as diagnostic tools for interpreting the risk map–MHSI mismatch, not as a complete causal pathway.

Relative to global and regional chikungunya or dengue risk models, the main strength of this framework is its ability to resolve neighborhood-scale heterogeneity within a single urban agglomeration. Those broader models remain valuable for mapping climatic suitability and enabling cross-regional comparison, but they often smooth over local variation within continuous built-up corridors and riverine wet environments. Compared with urban remote-sensing or machine-learning surveillance studies based on NDVI, NDWI, LST, rainfall, ovitrap observations, or vector indices, this study differs by using publicly reported sub-city outbreak locations and environmental background samples to calibrate a 10 m additive suitability index. Its contribution therefore lies in integrating three interpretive layers: a high-resolution environmental suitability surface, an external comparative reference surface, and human-process proxy layers represented by POIs together with OLS and GWR.

Although the two surfaces share a broad east-high and west-low pattern, they diverge locally because of differences in scale, covariates, modeling objectives, and possible human-process effects. The residual-difference field (risk map−MHSI) was therefore used as a diagnostic of representational mismatch: positive residuals indicate areas where the external reference assigns higher relative values than the local MHSI, whereas negative residuals indicate the opposite. Residual agreement suggests similar spatial emphasis between the two surfaces, while disagreement highlights locations where environmental suitability alone may not reproduce the broader comparative pattern.

**Figure 7:**
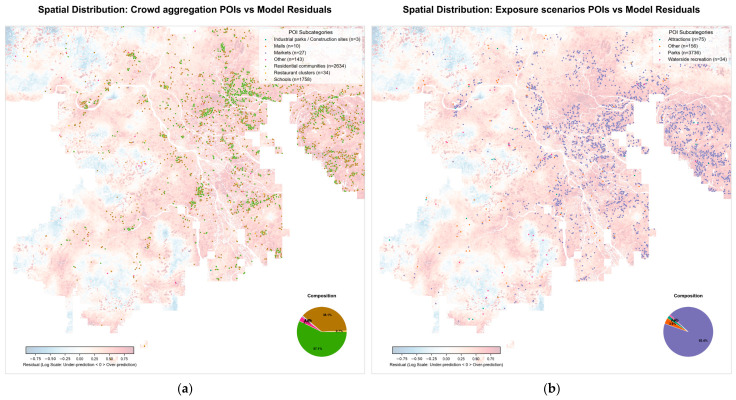

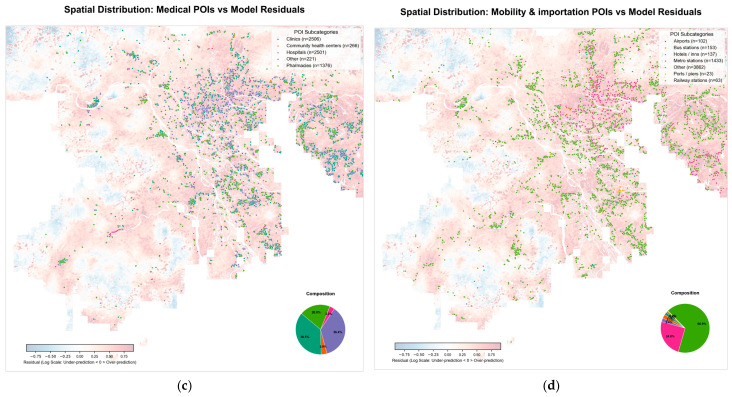
Overlay analysis between major POI groups and the residual-difference field: (**a**) crowd-aggregation POIs and residuals; (**b**) exposure-scenario POIs and residuals; (**c**) medical POIs and residuals; (**d**) mobility-and-importation POIs and residuals.

The POI overlays show that residual differences often coincide with organized urban activity spaces. Crowd-aggregation POIs, especially residential communities and schools, clustered in the eastern continuous built-up belt, while parks and other outdoor exposure settings were widely distributed across built-up and urban-rural transition zones. Medical and mobility-related POIs also showed spatial concentrations in several areas with elevated residuals. These patterns suggest that activity intensity, outdoor exposure, healthcare access, and transport-linked movement may contribute to spatial mismatch beyond environmental suitability, although the overlay itself remains descriptive.The spatial correspondence between major POI groups and the residual-difference field is shown in Figure 7.

To move beyond visual overlay, residual intensity and counts of major POI groups were aggregated to regular 3 km grid cells and analyzed using OLS and GWR. After log1p transformation, sparse-cell exclusion, and collinearity screening, the global OLS model showed positive associations for Medical (β = 0.2594, *p* < 0.001), Crowd Aggregation (β = 0.1083, *p* < 0.001), Mobility and Importation (β = 0.0933, *p* = 0.001), and Exposure scenarios (β = 0.0919, *p* < 0.001). However, its explanatory power was modest (R^2^ = 0.168; adjusted R^2^ = 0.166), indicating that a stationary relationship could not adequately capture the residual structure.

Allowing coefficients to vary spatially substantially improved model fit. The baseline GWR model achieved R^2^ = 0.6977 and adjusted R^2^ = 0.6627, with an automatically selected adaptive bandwidth of 122 grid cells, 1386 retained grid cells, and ENP = 143.49. To assess whether this improvement was driven by excessive local flexibility, we tested 2 km, 3 km, and 5 km grids and bandwidths equal to the automatically selected value and ±20%. Across the nine settings, R^2^ ranged from 0.6566 to 0.7492, adjusted R^2^ from 0.6250 to 0.7125, ENP from 63.05 to 276.27, and median local R^2^ from 0.4002 to 0.4804. Medical and Mobility/Importation coefficients were positive in most grid cells (Medical: 0.755–0.989; Mobility and Importation: 0.664–0.732), whereas Crowd Aggregation and Exposure scenarios were more spatially variable (Crowd Aggregation: 0.560–0.790; Exposure scenarios: 0.335–0.554). These results indicate that spatial non-stationarity was not dependent on a single grid or bandwidth setting, although local coefficient magnitudes and signs remain scale-sensitive.

**Figure 8:**
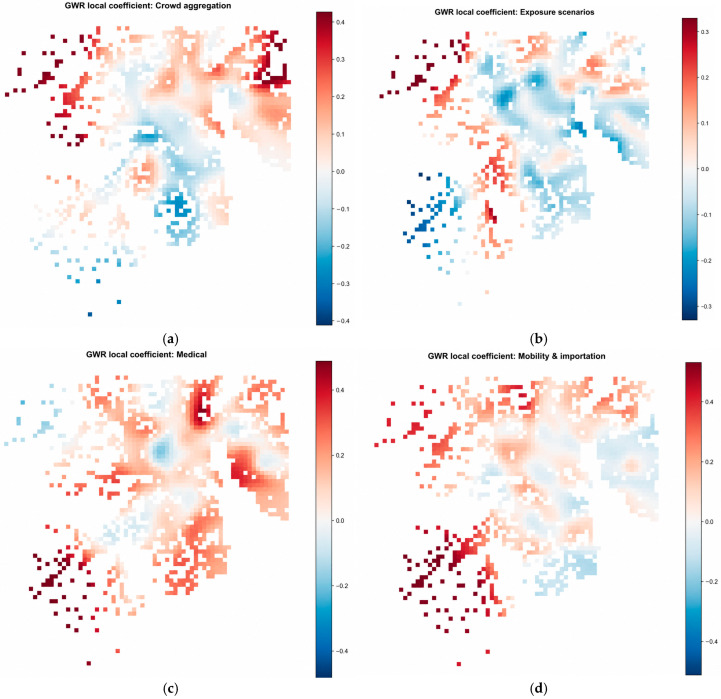
GWR results for major POI groups and residual intensity: (**a**) local coefficient surface for Crowd Aggregation, (**b**) local coefficient surface for Exposure scenarios, (**c**) local coefficient surface for Medical, (**d**) local coefficient surface for Mobility and Importation.

The local coefficient surfaces further show that POI-residual associations vary across the study area. Medical POIs were more strongly positive in several southern, eastern, and northeastern sectors, suggesting links with healthcare accessibility, reporting concentration, or the spatial organization of medical services. Mobility and Importation POIs showed stronger positive coefficients in southern and western corridor-like areas, consistent with transport-linked movement. Crowd Aggregation and Exposure-scenario POIs showed mixed signs, implying that residential, educational, commercial, recreational, and outdoor settings may operate differently across local urban contexts.The spatial heterogeneity of the GWR coefficients is shown in Figure 8.

Several limitations remain. The framework relies on publicly reported sub-city case locations and a single outbreak-period snapshot rather than a complete surveillance registry or extended time series. The internal assessment is not independent of the dataset used for weight tuning, and the external comparison relies on a model-derived risk map rather than an entomological survey surface or independent local surveillance benchmark. The sensitivity analyses suggest that the broad LST pattern and GWR interpretation are reasonably stable under alternative training-sample sizes, smoothing radii, grid sizes, and bandwidths, but uncertainty remains from public reporting, static March-May environmental composites, background sampling, and scale-dependent local coefficients. Future work should use more complete sub-city surveillance data, temporally resolved environmental conditions, and repeated outbreak periods before extending the conclusions beyond the 2025 Guangdong event.

## 5. Conclusions

This study developed a reproducible high-resolution workflow for assessing urban chikungunya environmental suitability and outbreak-related spatial heterogeneity in the western Guangdong–Hong Kong–Macao Greater Bay Area by integrating Sentinel-2 environmental indicators, a statistically refined 10 m land surface temperature surface, and a weighted additive MHSI. The resulting maps indicate a broad gradient of higher environmental suitability in the east and lower suitability in the west, with humidity- and water-related conditions contributing more strongly to the composite index than vegetation and temperature under the current model specification. Sensitivity analyses further indicated that the broad downscaled LST pattern and the exploratory GWR interpretation were not strongly dependent on the selected baseline training-sample size, residual-smoothing radius, grid scale, or bandwidth setting.

Internal consistency assessment showed that reported sub-city case locations were generally associated with higher MHSI values than randomly sampled background locations. Comparison with an external reference surface further revealed a moderate positive correspondence at the macro-spatial scale (Pearson’s r = 0.3421), and this association remained robust after correction for spatial autocorrelation using a modified t-test, torus-shift spatial null modeling, and block permutation. Because the same public case-location dataset contributed to weight calibration, however, the internal case-based comparison should be interpreted as a consistency check rather than as an independent validation.

The evidence presented here should therefore be regarded as preliminary rather than conclusive. The framework serves as a useful proof of concept for high-resolution environmental suitability mapping and exploratory interpretation of outbreak patterns, but it does not yet support operational prediction or causal inference for fine-scale intervention planning. Because it was developed using a single outbreak period and publicly reported sub-city case locations, its transferability to other cities, outbreak periods, or forecasting settings remains uncertain. Future work should incorporate more complete sub-city epidemiological data, explicitly model human-process variables, assess sensitivity to alternative sampling and spatial analysis choices, and evaluate the framework over longer temporal windows.

## Data Availability

The original contributions presented in this study are included in the article. Further inquiries can be directed to the corresponding author.

## Abbreviations

The following abbreviations are used in this manuscript:

CHIKV	Chikungunya virus
EVI	Enhanced Vegetation Index
LST	Land Surface Temperature
MHSI	Mosquito Habitat Suitability Index
NDBI	Normalized Difference Built-up Index
NDVI	Normalized Difference Vegetation Index
NDWI	Normalized Difference Water Index
H	Humidity Suitability
T	Temperature Suitability
V	Vegetation Suitability
W	Water Proximity
POI	Points of Interest
OLS	Ordinary Least Squares
GWR	Geographically Weighted Regression
RS	Remote Sensing
GEE	Google Earth Engine
AUC	Area Under the Receiver Operating Characteristic Curve
